# Simultaneous bright‐ and black‐blood whole‐heart MRI for noncontrast enhanced coronary lumen and thrombus visualization

**DOI:** 10.1002/mrm.26815

**Published:** 2017-07-19

**Authors:** Giulia Ginami, Radhouene Neji, Alkystis Phinikaridou, John Whitaker, René M. Botnar, Claudia Prieto

**Affiliations:** ^1^ Division of Imaging Sciences and Biomedical Engineering King's College London London United Kingdom; ^2^ MR Research Collaborations, Siemens Healthcare Limited Frimley United Kingdom; ^3^ Escuela de Ingeniería, Pontificia Universidad Católica de Chile Santiago Chile

**Keywords:** whole‐heart, bright‐blood, black‐blood, coronary MR angiography

## Abstract

**Purpose:**

To develop a 3D whole‐heart Bright‐blood and black‐blOOd phase SensiTive (BOOST) inversion recovery sequence for simultaneous noncontrast enhanced coronary lumen and thrombus/hemorrhage visualization.

**Methods:**

The proposed sequence alternates the acquisition of two bright‐blood datasets preceded by different preparatory pulses to obtain variations in blood/myocardium contrast, which then are combined in a phase‐sensitive inversion recovery (PSIR)‐like reconstruction to obtain a third, coregistered, black‐blood dataset. The bright‐blood datasets are used for both visualization of the coronary lumen and motion estimation, whereas the complementary black‐blood dataset potentially allows for thrombus/hemorrhage visualization. Furthermore, integration with 2D image‐based navigation enables 100% scan efficiency and predictable scan times. The proposed sequence was compared to conventional coronary MR angiography (CMRA) and PSIR sequences in a standardized phantom and in healthy subjects. Feasibility for thrombus depiction was tested ex vivo.

**Results:**

With BOOST, the coronary lumen is visualized with significantly higher (*P* < 0.05) contrast‐to‐noise ratio and vessel sharpness when compared to conventional CMRA. Furthermore, BOOST showed effective blood signal suppression as well as feasibility for thrombus visualization ex vivo.

**Conclusion:**

A new PSIR sequence for noncontrast enhanced simultaneous coronary lumen and thrombus/hemorrhage detection was developed. The sequence provided improved coronary lumen depiction and showed potential for thrombus visualization. Magn Reson Med 79:1460–1472, 2018. © 2017 International Society for Magnetic Resonance in Medicine. This is an open access article under the terms of the Creative Commons Attribution License, which permits use, distribution and reproduction in any medium, provided the original work is properly cited.

## INTRODUCTION

Coronary artery disease (CAD) primarily is caused by the formation and progressive growth of atherosclerotic plaque, which can lead to the narrowing of one or several of the coronary arteries and subsequent angina 1. Catheter‐based X‐ray coronary angiography remains the gold standard for the detection of CAD. However, a major challenge of CAD diagnosis remains that 50% of patients experiencing myocardial infarction have angiographically normal coronary arteries and no previous symptoms. In fact, plaque growth may occur with preserved coronary lumen size, also called “outward or positive arterial remodeling” 2, thus evading detection by luminographic imaging techniques. As the plaque increases, in size its tissue composition changes and may lead to plaque destabilization and eventually rupture, causing myocardial infarction or stroke due to occlusive thrombosis. The persistent presence of nonocclusive mural or intraluminal thrombosis also can cause sudden blockage and life‐threatening events through, for example, embolization. Recent studies have shown that coronary plaque burden, intraplaque hemorrhage, and thrombus carry significant prognostic value for the prediction of future coronary events 3. Thus, early detection of these features may allow to better risk stratify patients and ultimately improve patient outcome. Therefore, the ideal diagnostic test for CAD should simultaneously and noninvasively provide location/degree of lumen stenosis and detection/characterization of coronary plaque and thrombus.

MRI has shown great potential for noninvasive coronary lumen 4, thrombus/hemorrhage 5, and plaque visualization 3, 6, 7 in CAD patients. However, MR coronary lumen, thrombus/hemorrhage, and plaque assessment often suffers from image quality degradation due to physiological motion (during and between acquisitions) and prolonged scan times.

Coronary lumen visualization is achieved with bright‐blood coronary MR angiography (CMRA). 3D whole‐heart CMRA typically is performed with 1D diaphragmatic navigator gating and tracking to compensate for the respiratory motion of the heart 8. This approach leads to prolonged and unpredictable scan times because only a fraction of the acquired data (referred to as scan efficiency) is accepted for image reconstruction. Several advanced motion correction techniques have been proposed for CMRA during the last decade that allow the extraction of respiratory motion information directly from the imaging data, thus enabling 100% scan efficiency and more predictable scan times 9, 10, 11, 12, 13, 14, 15, 16, 17, 18, 19.

Characterization of coronary intraplaque hemorrhage and thrombus has been demonstrated using a 3D black‐blood noncontrast enhanced T_1_‐weighted inversion recovery (IR) sequence 5. Such approach exploits the short T_1_ of methemoglobin that is present in acute thrombus and intraplaque hemorrhage. Because the signal from the background tissues appears strongly suppressed in T_1_‐weighted black‐blood images, an additional bright‐blood CMRA needs to be acquired as anatomical reference; typically, the coronary lumen bright‐blood and the coronary thrombus/hemorrhage black‐blood acquisitions are performed sequentially. Similar sequential approaches have been successfully applied for coronary plaque characterization in patients with CAD 3, 7, 20.

Because the black‐blood and bright‐blood acquisitions usually are performed under free‐breathing, 1D diaphragmatic navigator gating and tracking 8 is used to account for respiratory motion. Aside from leading to prolonged and unpredictable scan times, as previously described for conventional CMRA, this sequential approach can lead to misregistration artifacts between the bright‐blood and black‐blood datasets. A 3D whole‐heart and respiratory self‐navigated radial sequence (CATCH) recently has been introduced, and it addresses some of these drawbacks by acquiring a black‐blood IR sequence and an anatomical bright‐blood reference in an alternate fashion 21. With this approach, however, the reconstruction scheme relies on respiratory motion parameters that are partially shared among the bright‐blood and the black‐blood data; as such, the risk of misregistration errors may be not entirely avoided. Furthermore, the absence of preparatory pulses prior to the acquisition of the bright‐blood anatomical reference scan may lead to suboptimal image contrast, in comparison with a dedicated CMRA sequence, thus reducing its diagnostic value.

In this study, we propose a novel 3D whole‐heart noncontrast enhanced Bright‐blood and black‐blOOd phase SensiTive (BOOST) IR sequence for simultaneous coronary lumen and coronary thrombus/intraplaque hemorrhage visualization. This is achieved by alternating the acquisition of two different bright‐blood whole‐heart datasets, which then are combined in a phase‐sensitive inversion recovery (PSIR)‐like reconstruction 22 to obtain a third, complementary, black‐blood dataset. The proposed approach 1) enables the acquisition of two differently weighted bright‐blood datasets carrying anatomical information (e.g., coronary lumen visualization), and from which respiratory motion independently can be estimated; 2) provides a coregistered black‐blood PSIR dataset for thrombus and hemorrhage visualization; and 3) introduces intrinsic robustness with respect to the choice of the inversion time (TI) by exploiting the PSIR framework. Furthermore, integration of the proposed sequence with 2D image‐based navigation 12 was pursued to enable 100% scan efficiency and predictable scan time. The proposed sequence was validated in a standardized phantom and compared to CMRA 4 with image‐based navigation 12 and PSIR sequences 22, 23 in healthy subjects. Furthermore, feasibility for thrombus depiction was tested ex vivo in a pig heart.

## METHODS

**Figure 1:**
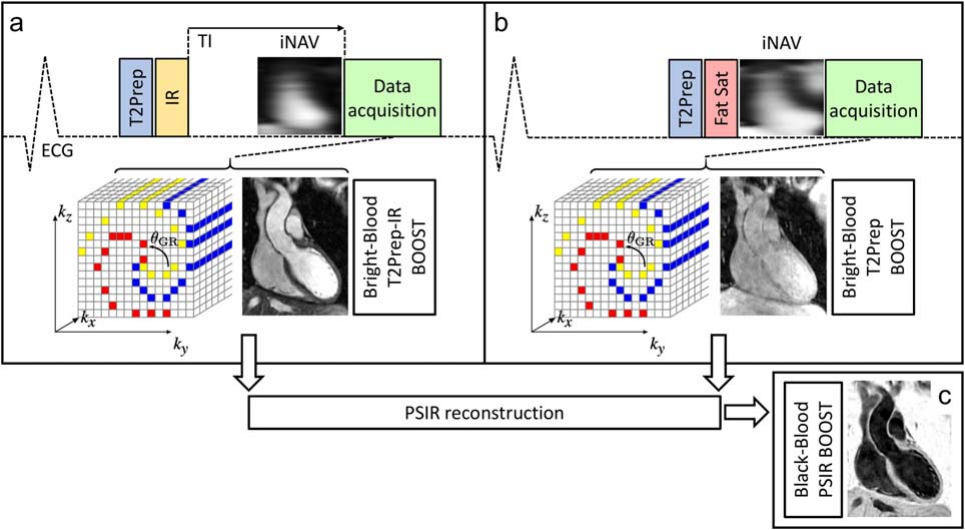
Proposed BOOST framework for simultaneous 3D whole‐heart noncontrast enhanced bright‐blood and black‐blood MR imaging for coronary lumen and thrombus visualization. A T_2_Prep‐IR module is applied at odd heartbeats (**a**) to enable coronary lumen visualization with improved contrast between blood and myocardium. A short TI is set to null the signal from epicardial fat. At even heartbeats (**b**), data acquisition is T_2_ prepared and performed with a high flip angle using spectral presaturation for fat suppression. Data acquisition is performed using a 3D Cartesian trajectory with spiral profile order 15 and is segmented over multiple heartbeats (yellow, red, blue) to minimize cardiac motion. A low‐resolution 2D iNAV 12 precedes the 3D data acquisition at each heartbeat and is used to estimate translational respiratory motion along the superior–inferior and right–left direction for both bright‐blood datasets (T_2_Prep‐IR BOOST and T_2_Prep BOOST) independently. The two motion‐corrected bright‐blood datasets are coregistered and then combined in a PSIR‐like reconstruction 22 to generate a 3D whole‐heart black‐blood dataset (PSIR BOOST) (**c**). BOOST, Bright‐blood and black‐blOOd phase SensiTive; ECG, electrocardiogram; iNAV, image‐based navigator; IR, inversion recovery; T_2_Prep, T_2_ prepared; PSIR, phase sensitive inversion recovery.

A previously published 3D whole‐heart, electrocardiogram‐triggered, balanced steady‐state free precession (bSSFP) Cartesian prototype sequence with spiral profile order 15 was extended to perform the BOOST acquisition, as shown in Figure 1. The proposed sequence alternates the acquisition of a bright‐blood T_2_‐prepared IR volume for optimized contrast between blood and myocardium (T_2_Prep‐IR BOOST, odd heartbeats) (Fig. 1a) and a bright‐blood T_2_‐prepared volume (T_2_Prep BOOST, even heartbeats) (Fig. 1b). For odd heartbeats, a short TI IR 24 approach is exploited to suppress fat signal (Fig. 1a), whereas spectral presaturation 25 was used to suppress signal from epicardial fat at even heartbeats (Fig. 1b). A 2D image‐based navigator (iNAV) 12 precedes each 3D whole‐heart bright‐blood data acquisition to enable beat‐to‐beat 2D translational respiratory motion estimation and compensation with 100% scan efficiency.

The two bright‐blood volumes (T_2_Prep‐IR BOOST (Fig. 1a) and T_2_Prep BOOST (Fig. 1b)) are then combined in a PSIR‐like reconstruction 22 to generate a PSIR BOOST black‐blood volume, using the T_2_Prep BOOST scan (even heartbeats) as the reference image for phase computation (Fig. 1c).

Respiratory motion detection and compensation are performed prior to the PSIR‐like reconstruction. Beat‐to‐beat 2D translational motion independently is estimated from the iNAVs for both T_2_Prep‐IR BOOST and T_2_Prep BOOST scans. A region of interest (ROI) for motion estimation is selected around the heart during acquisition planning. A template‐matching algorithm 26 is then utilized to estimate superior–inferior (SI) and right–left (RL) translational motion, using the first acquired iNAV of each dataset as respiratory reference position. Motion compensation is performed by modulating the k‐space data with a linear phase, corresponding to a rigid translational shift in the image domain as per the Fourier shift theorem. After beat‐to‐beat respiratory motion correction, and before PSIR‐like reconstruction, the two bright‐blood volumes are rigidly coregistered to the same respiratory position. Motion estimation and correction, image reconstruction, and PSIR‐like computation were implemented inline using the scanner software (Siemens Syngo MR E11A; Siemens Healthcare, Erlangen, Germany).

Phantom, healthy subjects, and ex vivo pig heart data acquisitions were performed on a 1.5T system (Magnetom Aera, Siemens Healthcare). Written informed consent was obtained from all subjects according to institutional guidelines, and the study was approved by the institutional review board. Prior to actual data acquisition, longitudinal magnetization behavior of myocardium and blood tissues induced by the proposed BOOST sequence was simulated to preliminarily assess blood/myocardium contrast in correspondence to both odd and even heartbeats.

### Sequence Simulation

The extended phase graphs (EPG) tool 27 was used to simulate both the BOOST sequence (as shown in Fig. 1) and a dedicated, T_2_‐prepared, CMRA acquisition. The simulation was performed considering the following tissue parameters: blood T_1_ = 1,490 ms; blood T_2_ = 245 ms; myocardial T_1_ = 800 ms; and myocardial T_2_ = 50 ms. T_1_ and T_2_ values of both blood and myocardium were set to match those mimicked by the standardized phantom used in this study and described in the following paragraphs 28. Similarly, imaging parameters were set equal to those of all the performed MRI acquisitions (phantom and in vivo). Data acquisition duration was set to 120 ms, corresponding to 33 k‐space lines acquired per heartbeat, which are preceded by 14 bSSFP ramp‐up pulses (corresponding to the iNAV). The simulated heart rate was 50 beats per minute, and 50 heartbeats were simulated in total.

### Phantom

#### Acquisition

**Figure 2:**
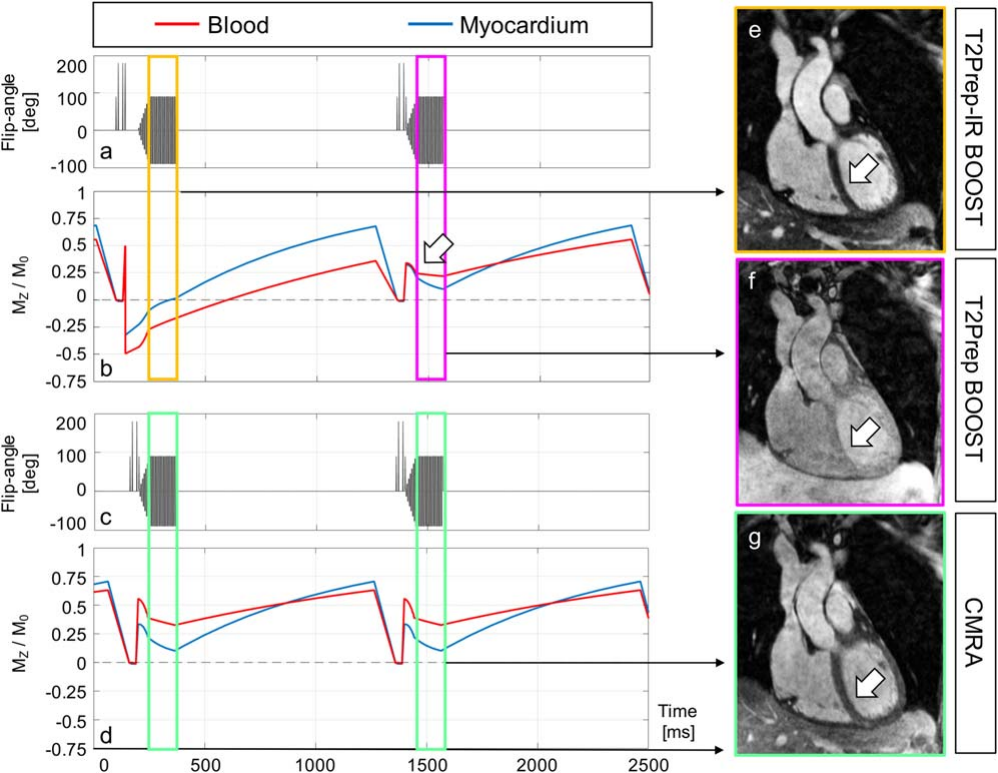
Simulated magnetization of the proposed BOOST sequence (**a**, **b**) and a more conventional and dedicated CMRA sequence (**c**, **d**) after steady state has been reached. The expected longitudinal magnetization (M_z_/M_0_) of myocardium (blue line) and blood (red line) is shown (**b**, **d**). With BOOST, the absolute signal ratio between blood and myocardium during data acquisition is optimized for odd heartbeats (ranging from 2.66 to 2.64, yellow rectangle). A strong contrast between the two tissues can be observed in the corresponding reconstructed T_2_Prep‐IR BOOST volume (**e**, arrow). Conversely, the absolute signal ratio between blood and myocardium is reduced in correspondence to the acquisition of even heartbeats (ranging from 1.35 to 2.11, purple rectangle). Specifically, such reduced signal ratio can be observed at the beginning of imaging data collection (arrow in **b**) and gets particularly emphasized with centric reordering acquisitions. As a consequence, reduced tissue contrast can be observed for the corresponding T_2_Prep BOOST reconstruction (**f**, arrow). Because T_2_Prep BOOST is used as the reference image in the process of PSIR reconstruction, reduced tissue contrast is indeed desired for adequate surface coil normalization and contrast of the resulting PSIR image. Conversely, and for the more conventional CMRA acquisition, the absolute blood to myocardium ratio ranges from 1.74 to 3.2 during data acquisition (green rectangles). Centric reordering acquisitions emphasize the improved blood to myocardium contrast with T_2_Prep‐IR BOOST in comparison to the dedicated CMRA. This can be appreciated in the corresponding image reconstructions (**e**, **g**, arrows). BOOST, Bright‐blood and black‐blOOd phase SensiTive; CMRA, conventional coronary MR angiography; T_2_Prep, T_2_ prepared.

Data were acquired in a standardized T_1_/T_2_ phantom 28 presenting several vials that mimic different cardiac compartments, including native blood (T_1_ = 1,490 ms, T_2_ = 245 ms), myocardium (T_1_ = 800 ms, T_2_ = 50 ms), and thrombus (T_1_ = 500 ms, T_2_ = 45 ms) (Fig. 2). Three different acquisitions were performed: 1) a conventional PSIR sequence 22 interleaving an IR pulse (odd heartbeats) and a low flip angle reference image without preparatory pulses (even heartbeats); 2) a previously published PSIR sequence 23 alternating the acquisition of a T_2_Prep‐IR scan (odd heartbeats) with a low flip‐angle reference image without preparatory pulses (even heartbeats); and 3) the proposed BOOST sequence, alternating the acquisition of a T_2_Prep‐IR scan (odd heartbeats) with a high flip angle T_2_‐prepared bright‐blood image (even heartbeats). These preliminary acquisitions were used for the evaluation of the signal measured in the different cardiac compartments with both BOOST and previously published PSIR sequences. Imaging parameters common to the three sequences were set as follows: transverse orientation, spatial resolution = 1 × 1 × 4 mm, field of view (FOV) = 320 × 320 × 96 mm, echo time (TE)/repetition time (TR) = 1.56/3.6 ms, pixel bandwidth 977 Hz/Pixel, and TI = 110 ms. For the sequences replicating the ones described in 22, 23, flip angles were set to 90 and 8 degrees for odd and even heartbeats, respectively. For the proposed BOOST sequence, the flip angle was 90 degrees for both odd and even heartbeats. The T_2_Prep duration for the sequences replicating the one described in 23 and for the proposed BOOST sequence was set to 40 ms.

#### Data Analysis

Signal‐to‐noise ratio (SNR) of blood (SNR_blood_) and contrast‐to‐noise ratio (CNR) between blood and myocardium (CNR_blood‐myo_) were measured for the images obtained with all the investigated sequences (conventional PSIR 22, T_2_Prep‐IR PSIR 23, and the proposed BOOST sequence) for both odd and even heartbeats scans. For all the black‐blood PSIR images, CNR_blood‐myo_ as well as CNR between thrombus and blood (CNR_th‐blood_) were quantified. A Wiener filter was used to remove low spatial frequency signal components before CNR quantifications in the PSIR images, as described in 22, 29.

### Healthy Subjects

#### Acquisition

A preliminary scan matching that of the phantom acquisition was performed in three healthy subjects to compare the image quality of the acquired iNAVs and demonstrate the performance of motion estimation/correction for all the investigated noncontrast enhanced PSIR sequences (conventional PSIR 22, T_2_Prep‐IR PSIR 23, and the proposed BOOST sequence). Data were acquired under free‐breathing in coronal orientation with the same parameters used for the phantom scan.

Data also were acquired in 11 additional healthy subjects (age 32.5 ± 7.6 years, 5 males, average heartrate ∼50 beats/minute) with the proposed BOOST sequence and a 3D whole‐heart CMRA acquisition integrated with image‐based navigation 12 for comparison purposes. For this cohort, BOOST data were acquired under free‐breathing with the following parameters: subject‐specific number of coronal slices covering the whole heart = 40–52, in‐plane resolution = 1 mm^2^, slice thickness = 2 mm, FOV = 320 × 320 × 80–104 mm, TE/TR = 1.56/3.6 ms, pixel bandwidth = 977 Hz/pixel, flip angle = 90°, T_2_ prep = 40 ms, and TI = 110 ms. Matching imaging parameters were employed for the dedicated CMRA scan. Nominal acquisition time for the proposed BOOST approach and for the CMRA scan was ∼18 and ∼9 minutes, respectively.

For all the acquisitions in healthy volunteers, a subject‐specific trigger delay was used coinciding with the mid‐diastolic rest period, and the acquisition window duration was chosen depending on the length of the identified quiescent period, ranging from 90 to 125 ms (corresponding to 25–35 k‐space lines acquired per heartbeat). All acquisitions were performed using a 2D iNAV for motion compensation with 100% scan efficiency. For the 2D iNAV acquisition, 14 bSSFP startup echoes (same FOV and orientation of the 3D acquisition, linearly increasing startup flip angles) were used. The rectangular ROI for tracking the respiratory motion of the heart was manually selected prior to data acquisition; such ROI was defined over the whole heart in RL direction, whereas it was covering the base and the midpart of the heart along the SI direction.

#### Data Analysis

The conventional PSIR 22, T_2_Prep‐IR PSIR 23, and proposed BOOST acquisitions, acquired in three healthy subjects, were visually compared in terms of image quality. In the cohort of 11 healthy subjects, the proposed BOOST (specifically the T_2_Prep‐IR BOOST bright‐blood volume) and the conventional CMRA acquisitions were compared in terms of coronary lumen visualization using quantitative parameters including SNR, CNR, vessel length, and sharpness. Specifically, visible coronary vessel length and coronary percentage vessel sharpness (%VS) for both the right coronary artery (RCA) and the left anterior descending (LAD) coronary artery were measured for the bright‐blood T_2_Prep‐IR BOOST dataset before and after motion correction, and for the conventional CMRA scan after motion correction only. Quantification of coronary %VS was performed for the entire length of the coronary segment of interest, as well as for the first visible 4 cm using the software described in 30. SNR_blood_ and CNR_blood‐myo_ were computed for the bright‐blood T_2_Prep‐IR BOOST dataset after motion correction and for the conventional CMRA scan. Furthermore, CNR_blood‐myo_ was quantified in the black‐blood PSIR BOOST images, after the removal of low spatial frequency signal components. A ROI characterized by uniform signal intensity was selected outside the body of the subject for the quantification of the standard deviation of the background signal. For all quantified endpoints, statistical analysis was performed using a paired two‐tailed Student *t* test and considering *P* = 0.05 as the threshold for statistical significance.

### Ex Vivo Pig Heart Thrombus

#### Acquisition

Following euthanasia, a pig heart was extracted. The left ventricular cavity was rinsed with saline prior to being packed with sand. Blood was not completely removed from the right ventricular cavity where it had coagulated. The heart was fixed in formalin and imaged 4 weeks later to demonstrate the feasibility for thrombus visualization. Data acquisition was performed with the proposed BOOST sequence, and with imaging parameters that matched those of the in vivo acquisitions. Furthermore, 2D T_1_ and T_2_ maps were acquired. For the T_1_ mapping acquisition, a 5‐3‐3 modified look‐locker inversion recovery sequence 31 was used with the following relevant imaging parameters: 5 slices; bSSFP readout; in‐plane resolution = 1.4 mm^2^; slice thickness = 8 mm; slice gap = 1.6 mm; FOV = 360 × 200 × 80 mm; pixel bandwidth = 1,085 Hz/pixel; and flip angle = 35°. The bSSFP sequence alternating three different T_2_Prep times for T_2_ mapping 32 was performed with the following imaging parameters: in‐plane resolution = 1.9 mm^2^; slice thickness = 8 mm; slice gap = 1.6 mm; FOV = 360 × 200 × 80 mm; pixel bandwidth = 1,185 Hz/pixel; flip angle = 70°.

#### Data Analysis

The bright‐blood T_2_Prep‐IR BOOST images in the ex vivo pig heart were evaluated in terms of CNR between the cavities and the myocardium. Furthermore, in the black‐blood BOOST image, CNR between thrombus and cavities and CNR between thrombus and myocardium were computed. Two regions of interest were manually selected in both the T_1_ and the T_2_ maps, at the level of the myocardium and of the thrombus, to quantify T_1_ and T_2_ of those compartments.

## RESULTS

Sequence simulation, data acquisition, and reconstruction were carried out successfully, and all the quantified endpoints are reported hereafter.

### Sequence Simulation

The simulated pulse sequences (BOOST sequence and dedicated CMRA) and the resulting behavior of the steady state magnetization of myocardium and blood are displayed in Figure 2. For the BOOST sequence (Figs. 2a,b), and in correspondence to odd heartbeats (T_2_Prep‐IR BOOST), the expected magnetization M_z_/M_0_ of blood varied between −0.2741 and −0.1452 during image acquisition, whereas the expected magnetization M_z_/M_0_ of the myocardium varied between −0.1028 and 0.0548, with zero crossing occurring during the interval of imaging data collection (yellow rectangle). This resulted in an almost constant absolute signal intensity ratio, varying from 2.66 to 2.64, between blood and myocardium. In even heartbeats (T_2_Prep BOOST), the expected magnetization M_z_/M_0_ of blood varied between 0.2344 and 0.2195, whereas the expected magnetization M_z_/M_0_ of the myocardial signal varied between 0.1731 and 0.1038. This resulted in a reduced contrast between blood and myocardium, and a reduced absolute signal intensity ratio ranging between 1.35 and 2.11 (purple rectangle). Because the k‐space center—carrying information on the resulting imaging contrast—is collected at the beginning of the acquisition window (centric reordering, as with the proposed approach, arrow in Fig. 2b), the resulting contrast ratio between myocardium and blood is particularly reduced. The sequence simulation for precontrast imaging thus suggests that the T_2_Prep‐IR BOOST dataset (odd heartbeats; Fig. 2e, arrow) should provide improved contrast and coronary delineation in comparison to the T_2_Prep BOOST dataset (even heartbeats; Fig. 2f, arrow). In addition, the reduced tissue contrast in the T_2_Prep BOOST dataset, which is used as reference image for PSIR reconstruction, is desirable for surface coil normalization and does not alter the final contrast of the normalized PSIR image 22. For the more conventional and dedicated CMRA acquisition (Figs. 2c,d), the expected magnetization M_z_/M_0_ of the blood varied between 0.3857 and 0.3280, whereas the expected magnetization M_z_/M_0_ of the myocardial signal varied in the interval 0.2214–0.1025; this resulted in an absolute signal intensity ration between blood and myocardium ranging from 1.74 to 3.2 during the interval of imaging data collection. Centric reordering acquisitions emphasize the improved contrast between myocardium and blood with T_2_Prep‐IR BOOST with respect to the dedicated CMRA acquisition (Fig. 2e vs. Fig. 2g, arrows).

### Phantom

**Figure 3:**
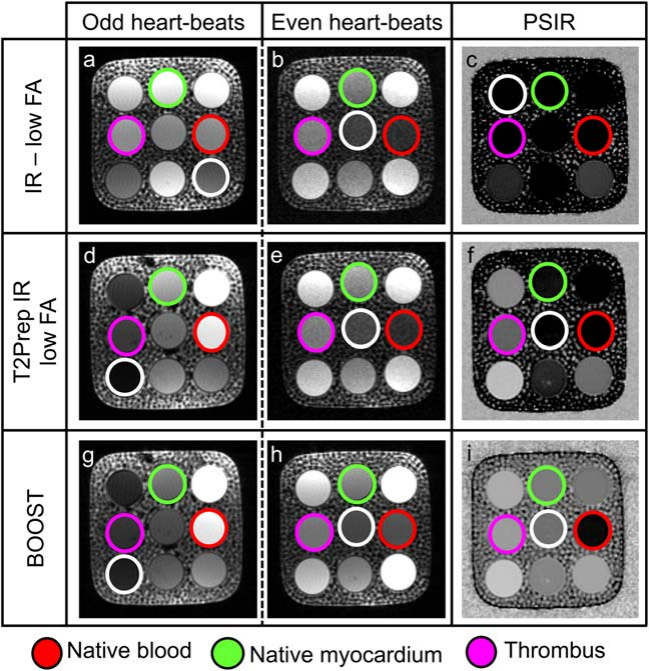
Data acquisition in a standardized T_1_/T_2_ phantom using three different sequences: the PSIR sequence described in 22, alternating the acquisition of a T_1_‐weighted volume with the acquisition of a reference image acquired at low flip angle and without preparatory pulses (first row); the T_2_Prep‐IR PSIR sequence described in 23 that alternates the acquisition of a volume preceded by a T_2_Prep‐IR module with a reference acquisition performed at low FA angle and without preparatory pulses (second row); and the proposed BOOST sequence illustrated in Figure 1 (third row). Compartments of interest are indicated: blood (red circle), myocardium (green circle), and thrombus (purple circle). For all the images, SNR_blood_ as well as CNR_blood‐myo_ were computed with respect to a low‐signal compartment (white circle). With all the investigated sequences, the images acquired at odd heartbeats exhibit high SNR_blood_ (**a**, **d**, **g**) and high CNR_blood‐myo_. Due to the relatively short T_1_ of the vial mimicking the coronary thrombus (purple circle), its signal appears suppressed in the images acquired at odd heartbeats with all the investigated sequences. The images (**b**) and (**e**), acquired at even heartbeats, show low SNR because data acquisition is performed at low FAs. Differently, higher SNR_blood_ and CNR_blood‐myo_ were observed with the proposed BOOST approach because data acquisition was performed with a 90‐degree FA. The PSIR reconstruction provided in (**c**) shows signal suppression for all the compartments of interest; differently, in the PSIR‐like reconstructions displayed in (**f**) and (**i**), a black‐blood effect is obtained, with remarkable depiction of the vial mimicking the thrombus. BOOST, Bright‐blood and black‐blOOd phase SensiTive; FA, flip angle; IR, inversion recovery; myo, myocardium; PSIR, phase sensitive inversion recovery; T_2_Prep, T_2_ prepared.

Conventional PSIR 22, T_2_Prep‐IR PSIR 23, and proposed BOOST (first, second, and third row, respectively) phantom acquisitions are shown in Figure 3 for odd heartbeats (first column), even heartbeats (second column), and black‐blood PSIR‐like reconstruction (third column) for an inversion time of 110 ms (optimized for the noncontrast enhanced BOOST sequence). Vials corresponding to native blood (red circle), native myocardium (green circle), and thrombus signal (purple circle) are shown in Figure 2, together with a low‐signal compartment (white circle) used to quantify the background noise in each image. All the quantified values for SNR_blood_, CNR_blood‐myo_, and CNR_th‐blood_ are reported in Supporting Table S1. Insufficient CNR_blood‐myo_ and CNR_th‐myo_ were measured for the PSIR reconstruction obtained from the sequence resembling that in 22, which has been introduced for postcontrast acquisitions with typical inversion times ∼200 ms. More similar contrasts were provided by the sequence resembling that in 23 (also introduced with typical inversion times ∼200 ms) and the proposed BOOST approach in correspondence to odd heartbeats and in the PSIR reconstruction. Furthermore, and consistent with the sequence EPG simulation, data acquired at odd heartbeats with the proposed approach (T_2_Prep‐IR BOOST) showed better SNR_blood_ and CNR_blood‐myo_ if compared to data acquired at even heartbeats (T_2_Prep BOOST); therefore, the T_2_Prep‐IR BOOST dataset was selected for noncontrast enhanced coronary lumen visualization in the healthy volunteers and ex vivo pig heart studies.

### Healthy Volunteers

Image‐based navigation allowed data acquisition at 100% scan efficiency, and none of the acquired imaging data was discarded in the process of image reconstruction.

**Figure 4:**
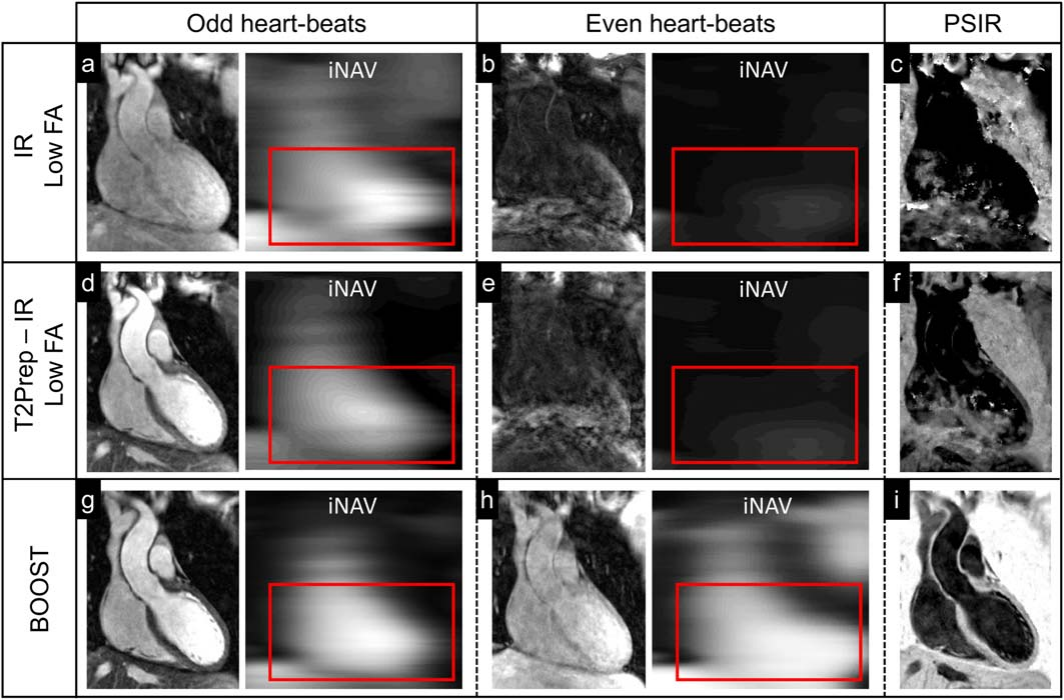
Data acquisition in a healthy subject using the same sequences as for the phantom acquisition: conventional PSIR 22 (first row), T_2_Prep‐IR PSIR 23 (second row), and the proposed BOOST approach (third row). For odd heartbeats acquisitions, the high contrast on the preceding iNAV allows for adequate motion tracking in all cases (**a**, **d**, **g**). For even heartbeats acquisitions, however, the low signal of the image and the corresponding iNAV in (**b**) and (**e**) prevent successful motion estimation. This results in registration errors and inadequate contrast in the derived PSIR image (**c**, **f**). With the proposed BOOST sequence, however, motion information can be accurately extracted for even heart beats as well, thanks to the higher signal of the imaging data and the high contrast of the iNAVs (**h**). Therefore, a sharply defined black‐blood dataset can be obtained from the PSIR‐like computation (**i**). BOOST, Bright‐blood and black‐blOOd phase SensiTive; FA, flip angle; iNAV, image navigator; IR, inversion recovery; PSIR, phase‐sensitive inversion recovery; T_2_Prep, T_2_ prepared.

Figure 4 shows images from the conventional PSIR 22 (first row), T_2_Prep‐IR PSIR 23 (second row), and proposed BOOST (third row) for a representative healthy subject and for odd heartbeats (first column), even heartbeats (second column), and black‐blood PSIR‐like reconstruction (third column). Imaging data and corresponding iNAVs for the conventional PSIR and T_2_Prep‐IR PSIR acquisitions in even heartbeats (Figs. 4b and 4e) showed very low contrast. The low‐contrast iNAVs led to inaccurate respiratory motion estimation and to severe artifacts in the derived noncontrast enhanced PSIR reconstruction (Figs. 4c and 4f). In contrast, the proposed BOOST sequence ensured adequate respiratory motion estimation for both bright‐blood acquisitions (odd and even heart beats, Figs. 4g and 4h, respectively), resulting in a sharply defined noncontrast enhanced black‐blood PSIR‐like reconstruction (Fig. 4i).

**Figure 5:**
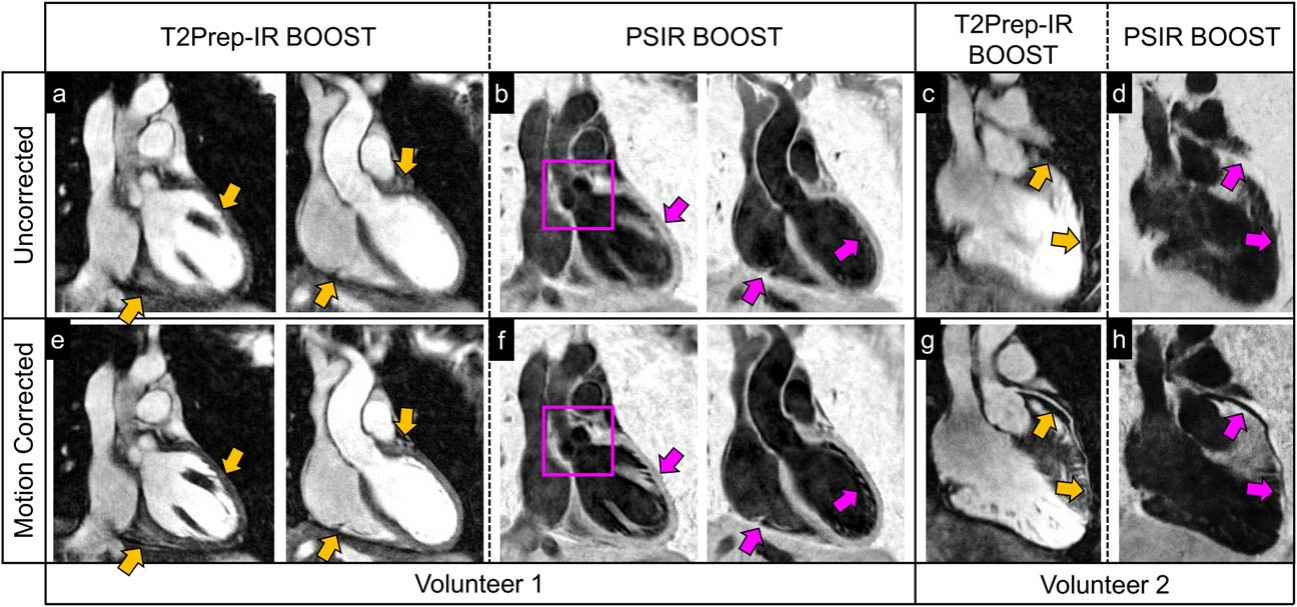
Image quality improvement after motion correction in two healthy volunteers with the proposed BOOST framework. Bright‐blood T_2_Prep‐IR BOOST and black‐blood PSIR BOOST images are shown before (first row) and after (second row) motion correction in acquired coronal orientation (volunteer 1) and reformatted plane (volunteer 2). Yellow arrows show visible improvement in image quality at the level of the myocardium and of the interface between the heart and the liver for the motion‐corrected T_2_Prep‐IR BOOST dataset (**a** versus **e**). Similarly, a sharper depiction of anatomical structures (purple arrows), including the aortic valve (purple square), can be appreciated in the motion‐corrected black‐blood PSIR BOOST images (**f**) when compared to the uncorrected ones (**b**). For the second volunteer, motion correction successfully recovers a sharp visualization of the left anterior descendent coronary artery (LAD) for both the bright‐blood T_2_Prep‐IR BOOST dataset (**c** and **g**, yellow arrows) and the black‐blood PSIR BOOST dataset (**d** and **h**, purple arrows). BOOST, Bright‐blood and black‐blOOd phase SensiTive; IR, inversion recovery; PSIR, phase‐sensitive inversion recovery; T_2_Prep, T_2_ prepared.

T_2_Prep‐IR bright‐blood and black‐blood BOOST images are shown before (first row) and after motion correction (second row) in Figure 5 for two representative volunteers. With the proposed BOOST sequence, 2D translational motion correction leads to a reduction of motion artifacts and an improvement in coronary delineation for the bright‐blood images and the black‐blood reconstruction.

**Figure 6:**
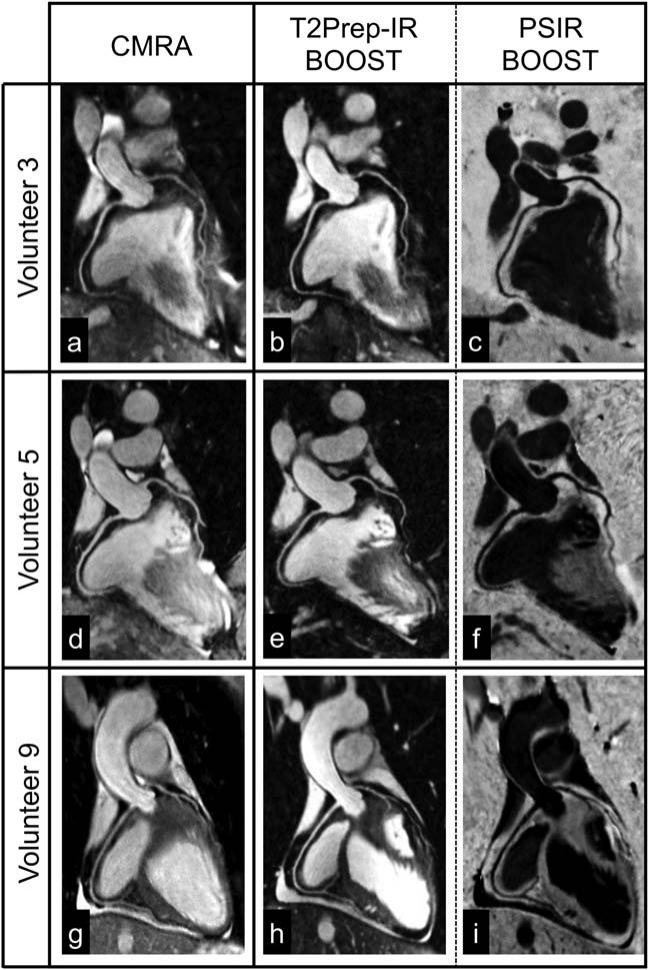
Reformatted coronary depiction in three representative healthy volunteers obtained with a conventional T_2_‐prepared bright‐blood CMRA acquisition (**a**, **d**, **g**) and the proposed BOOST sequence for simultaneous bright‐blood (T_2_Prep‐IR BOOST datasets in **b**, **e**, **h**) and black‐blood (PSIR BOOST datasets in **c**, **f**, **i**) whole‐heart MRI. Quantified CNR_blood‐myo_ significantly improved with the proposed T_2_Prep‐IR BOOST approach in comparison to the conventional CMRA, thus leading to a higher quantified coronary percentage vessel sharpness (%VS) for both right and left coronary arteries. In the PSIR BOOST images in (**c**, **f**, **i**), the efficacy of blood signal suppression can be appreciated along multiple portions of the coronary tree. BOOST, Bright‐blood and black‐blOOd phase SensiTive; CMRA, conventional coronary MR angiography; IR, inversion recovery; myo, myo, myocardium; PSIR, phase‐sensitive inversion recovery; T_2_Prep, T_2_ prepared.

Bright‐blood T_2_Prep‐IR and black‐blood PSIR BOOST images, reformatted along the right and left coronary arteries, are shown in Figure 6 for three representative volunteers, in comparison to the conventional CMRA approach.

For the cohort of 11 healthy subjects, motion correction led to a statistically significant improvement (*P* < 0.05) in terms of visible coronary vessel length and %VS (Table 1). After 2D translational motion correction, coronary visible vessel length improved from 7.03 ± 1.50 cm to 8.58 ± 2.27 cm and from 4.82 ± 3.78 cm to 7.92 ± 4.16 cm for RCA and LAD, respectively, in the T_2_Prep‐IR BOOST images. Similarly, coronary %VS (for the first 4 visible cm) improved from 36.54% ± 8.63% to 48.31% ± 6.71% and from 32.92% ± 16.08% to 48.82% ± 6.51% after motion correction for the RCA and LAD, respectively. Coronary %VS along the entire coronary artery improved from 37.96% ± 8.63% to 48.82% ± 4.63% for the RCA, and from 32.42% ± 15.15% to 48.12% ± 6.63% for the LAD. All quantified endpoints related to the comparison between the dedicated CMRA and the motion‐corrected T_2_Prep‐IR BOOST are reported in Table 2. Quantified coronary visible vessel length for the T_2_Prep‐IR BOOST volume was similar to that of the conventional CMRA acquisition for the LAD (7.92 ± 4.16 vs. 7.17 ± 3.22, *P* = NS), whereas it was significantly higher for the RCA (8.58 ± 2.27 vs. 7.89 ± 1.77, *P* < 0.05). Quantified coronary %VS was found to be significantly higher (*P* < 0.05 in all cases) in the T_2_Prep‐IR BOOST volumes when compared to the conventional CMRA acquisition. %VS of the RCA increased from 43.70% ± 7.93% to 48.31% ± 6.71% (first 4 cm) and from 42.38% ± 6.29% to 48.82% ± 4.63% (entire vessel length). Furthermore, %VS of the LAD increased from 43.70% ± 7.93% to 48.82% ± 6.51% (first 4 cm) and from 42.38% ± 6.29% to 48.12% ± 6.63% (entire vessel length). T_2_Prep‐IR BOOST datasets led to higher CNR_blood‐myo_ and comparable SNR_blood_ when compared to the conventional CMRA acquisition, thus providing coronary depiction with improved delineation and higher contrast (Fig. 5). SNR_blood_ was 20.15 ± 8.63 for the T_2_Prep‐IR BOOST dataset and 22.35 ± 6.87 for the conventional CMRA acquisition (P = NS). CNR_blood‐myo_ increased to 14.89 ± 6.51 for the T_2_Prep‐IR BOOST compared to 12.24 ± 4.96 for the dedicated CMRA sequence, respectively (*P* < 0.025). CNR_blood‐myo_ (signal myocardium > signal blood) in the black‐blood BOOST images was 13.5 ± 23.7, with the signal of the myocardium being 17.7% ± 2.5% higher than that of blood.

**Table 1:** Quantitative Comparison Between Uncorrected and Motion‐Corrected T_2_Prep‐IR BOOST Datasets.

		Uncorrected T_2_Prep‐IR BOOST	Motion Corrected T_2_Prep‐IR BOOST	
RCA	Length (cm)	7.03 ± 1.50	8.58 ± 2.27	*P* < 0.02
	%VS (4 cm)	36.54 ± 8.63	48.31 ± 6.71	*P* < 0.001
	%VS (tot)	37.96 ± 8.63	48.82 ± 4.63	*P* < 0.001
LAD	Length (cm)	4.82 ± 3.78	7.92 ± 4.16	*P* < 0.02
	%VS (4 cm)	32.92 ± 16.08	48.82 ± 6.51	*P* < 0.005
	%VS (tot)	32.42 ± 15.15	48.12 ± 6.63	*P* < 0.005

Coronary visible vessel length and %VS are reported for both LAD and RCA.

BOOST, Bright‐blood and black‐blOOd phase SensiTive; IR, inversion recovery; LAD, left anterior descending; RCA, right coronary artery; T_2_Prep, T_2_ prepared; VS, vessel sharpness.

**Table 2:** Quantitative Comparison Between Conventional CMRA and Motion‐Corrected T_2_Prep‐IR BOOST Datasets.

		CMRA	Motion Corrected T_2_Prep‐IR BOOST	
RCA	Length (cm)	7.89 ± 1.77	8.58 ± 2.27	*P* < 0.05
	%VS (4 cm)	43.70 ± 7.93	48.31 ± 6.71	*P* < 0.005
	%VS (tot)	42.38 ± 6.29	48.82 ± 4.63	*P* < 0.001
LAD	Length (cm)	7.17 ± 3.22	7.92 ± 4.16	*P* = NS
	%VS (4 cm)	43.70 ± 7.93	48.82 ± 6.51	*P* < 0.001
	%VS (tot)	42.38 ± 6.29	48.12 ± 6.63	*P* < 0.001
SNR _blood_		22.35 ± 6.87	20.15 ± 8.63	*P* = NS
CNR _blood‐myo_		12.24 ± 4.96	14.89 ± 6.51	*P* < 0.025

Coronary visible vessel length and %VS are reported for both LAD and RCA, together with SNR_blood_ and CNR_blood‐myo_.

BOOST, Bright‐blood and black‐blOOd phase SensiTive; CMRA, conventional coronary MR angiography; CNR, contrast‐to‐noise ratio; IR, inversion recovery; LAD, left anterior descending; myo, myocardium; RCA, right coronary artery; SNR, signal‐to‐noise ratio; T_2_Prep, T_2_ prepared; VS, vessel sharpness.

### Ex Vivo Pig Heart Thrombus

**Figure 7:**
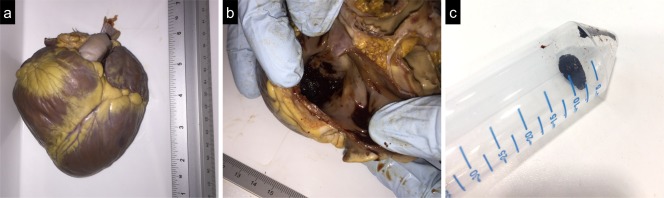
Ex vivo pig heart (**a**) presenting a blood clot at the level of the right ventricle (**b**) and used as a model in this study. The pig heart was fixed in formalin, and sand was used to fill the cavities. A portion of the blood clot was extracted, and it is here depicted for visualization purposes (**c**).

A section of the ex vivo pig heart providing thrombus visualization is reported in Figure 7, together with a small portion of the blood clot extracted from the bigger thrombus.

**Figure 8:**
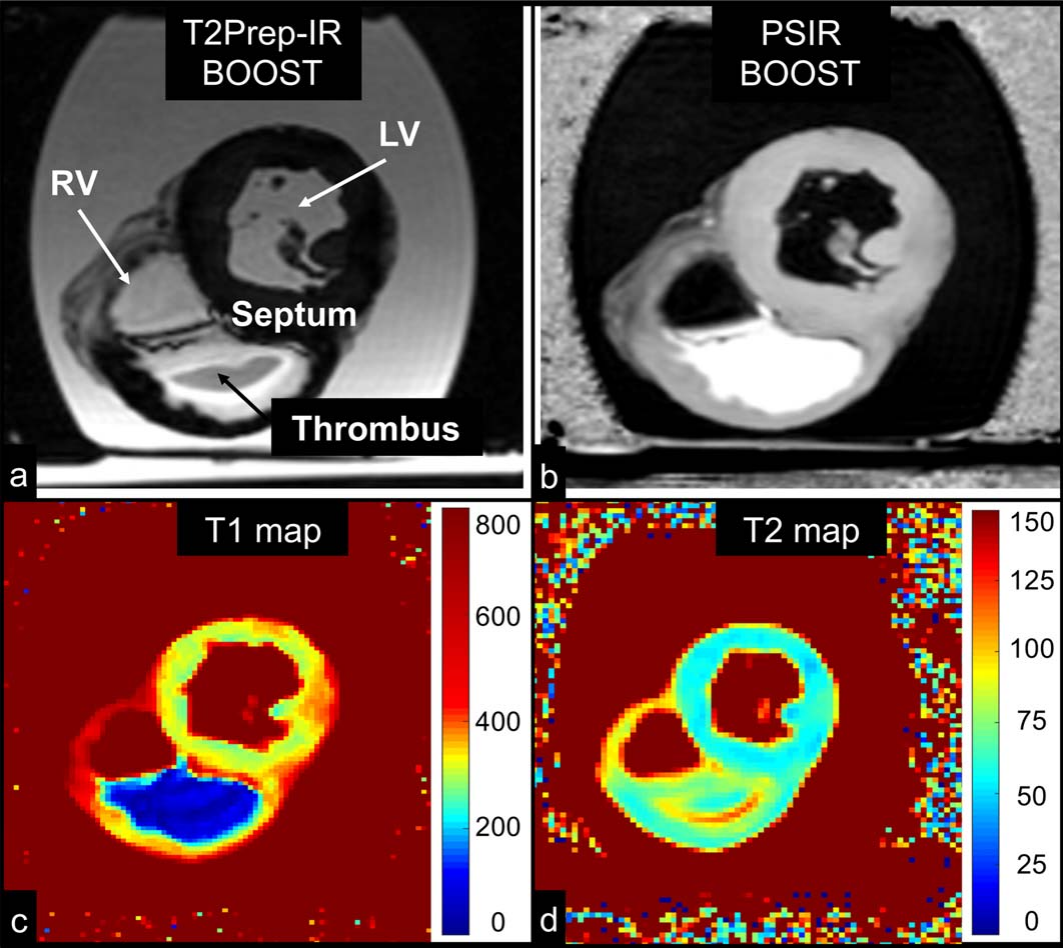
MRI images obtained in the ex vivo pig heart. All the images depict a short‐axis view at the midventricular level. Images acquired with the proposed BOOST sequence are reported in (**a**) (bright‐blood T_2_Prep‐IR dataset) and in (**b**) (black‐blood PSIR‐like reconstruction). RV, LV, thrombus, and interventricular septum are indicated. The black‐blood reconstruction (**b**) clearly enhances the signal from the thrombus when compared to the bright‐blood dataset (**a**). Furthermore, 2D T_1_ (**c**) and T_2_ (**d**) mapping sequences were acquired. The ex vivo thrombus is characterized by a relatively short T_1_ and T_2_. BOOST, Bright‐blood and black‐blOOd phase SensiTive; IR, inversion recovery; LV, left ventricular cavity; PSIR, phase‐sensitive inversion recovery; RV, right ventricular cavity; T_2_Prep, T_2_ prepared.

The bright‐blood T_2_Prep‐IR and black‐blood BOOST images obtained from the ex vivo pig heart are shown in Figures 8a and 8b, together with corresponding T_1_ and T_2_ maps (Figs. 8c,d). The black‐blood BOOST image shows good thrombus depiction with hyperintense signal (Fig. 8b). Signal from the thrombus was higher than the signal from the myocardium (+12.4%) and the signal from the cavities (+41.2%). Quantified CNR between thrombus and myocardium was 30.0, whereas it amounted to 79.3 between thrombus and cavities. Quantified T_1_ and T_2_ values for the compartments of interest amounted to T_1_ = 60.2 ± 23.4 ms and T_2_ = 73.0 ± 8.6 ms for the thrombus and T_1_ = 313.6 ± 22.2 ms and T_2_ = 56.0 ± 1.8 ms for the myocardium.

## DISCUSSION

### Technical Development

This study introduces a novel 3D whole‐heart noncontrast enhanced PSIR sequence for simultaneous coronary lumen and potential thrombus/intraplaque hemorrhage visualization. This is achieved by alternating a bright‐blood T_2_‐prepared IR (T_2_Prep‐IR BOOST, odd heartbeats) and a bright‐blood T_2_‐prepared (T_2_Prep BOOST, even heartbeats) whole‐heart acquisition, which then are combined in a PSIR‐like reconstruction 22 to obtain a complementary black‐blood volume (PSIR BOOST). The two bright‐blood volumes that are obtained with BOOST are differently weighted to provide different contrasts between myocardium and blood (Fig. 2). Contrast is enhanced for the T_2_Prep‐IR BOOST dataset, which is used for improved coronary lumen visualization. Conversely, tissue contrast in even heartbeats (T_2_Prep BOOST) is reduced to obtain adequate surface coil intensity normalization during PSIR reconstruction and to not significantly affect the contrast of the resulting normalized PSIR image 22. In contrast to previous PSIR implementations for late gadolinium enhanced myocardial infarction detection 22, 33, 34, 35 or 2D black‐blood coronary vessel wall imaging 36, the proposed technique provides whole‐heart coverage with the potential for simultaneous noncontrast enhanced coronary lumen and thrombus/hemorrhage visualization.

The entire BOOST framework was integrated with image‐based navigation 12 to compensate for 2D translational respiratory motion while ensuring 100% scan efficiency (no need for data rejection), thus enabling predictable scan time. The acquisition of two different bright‐blood datasets (T_2_Prep‐IR BOOST and T_2_Prep BOOST) preceded by iNAVs ensures that respiratory motion information independently can be extracted from the two datasets. The presence of rapid spatial variations in the reference image that is used for phase computation in a PSIR reconstruction may cause artifacts and reduced SNR, and also may lead to incorrect clinical interpretation of the resulting PSIR image 22. For this reason, it is important to ensure an optimal degree of motion compensation in both the magnitude (T_2_Prep‐IR BOOST) and the reference image (T_2_Prep‐BOOST), as well as accurate coregistration between the two datasets. The use of a PSIR‐like reconstruction to obtain the complementary black‐blood dataset introduces intrinsic robustness with respect to the choice of the TI; this might be particularly advantageous in the case of heart rate irregularities and in all the cases where the prediction of an optimal TI remains particularly challenging 37. When compared to previously published PSIR approaches 22, 23, which alternate the acquisition of an IR or a T_2_Prep‐IR acquisition with a low flip angle (without preparatory pulses) reference scan, the proposed BOOST sequence seems to be more effective in terms of respiratory motion estimation. A qualitative comparison between BOOST and more conventional PSIR sequences 22, 23 showed that the extraction of respiratory motion from the reference dataset acquired with low flip angle might be unreliable due to the poor signal of the preceding iNAV (Fig. 4). This resulted in poor image quality of the PSIR reconstructions. In contrast, motion estimation with BOOST was successful for both odd and even heartbeats, and adequate black‐blood contrast could be restored. In this study, a comparison between the proposed BOOST approach and previously published PSIR sequences was performed in only three healthy subjects and for illustrative purposes. Although it is reasonable to speculate that respiratory motion correction is more effective when respiratory motion is extracted from both odd and even heartbeats, more extensive studies are now warranted to corroborate our preliminary findings. Furthermore, the imaging parameters that were used for performing data acquisition with both conventional PSIR 22 and T_2_Prep‐IR PSIR 23 were set to match those of the optimized BOOST sequence for comparison purposes. However, imaging parameters leading to optimized contrast with BOOST may be suboptimal for other PSIR sequences, which conventionally are used for postcontrast acquisitions; thus, further and more extensive studies are required to compare the proposed BOOST sequence with previously published PSIR approaches.

Previously published approaches for thrombus imaging 5 or plaque assessment 3, 7, 20 rely on the sequential acquisition of a black‐blood volume and a bright‐blood anatomical reference and thus render image fusion challenging. The more recently published CATCH sequence 21 instead provides two imaging volumes with different contrasts that are acquired in an interleaved fashion. In contrast, the proposed BOOST framework provides complementary diagnostic information by performing two different reconstructions of the same 3D whole‐heart volume (magnitude reconstruction of T_2_Prep‐IR BOOST for bright‐blood coronary lumen visualization and PSIR reconstruction of T_2_Prep‐IR BOOST for black‐blood thrombus visualization). Although this may lead to improved image registration between the bright‐blood and the black‐blood volume, future studies should aim at a more exhaustive comparison between BOOST and prepublished T_1_‐weighted sequences.

Preliminary results in a standardized phantom and using EPG sequence simulation clearly showed that data acquired at odd heartbeats (T_2_Prep‐IR BOOST) provide improved blood SNR and CNR between native blood and myocardium when compared to data acquired at even heartbeats (T_2_Prep BOOST); consequently, the T_2_Prep‐IR BOOST dataset was selected for quantitative comparison against conventional CMRA. In a cohort of 11 healthy subjects, T_2_Prep‐IR BOOST showed improved CNR between blood and myocardium, as well as improved coronary %VS in comparison with the dedicated CMRA acquisition. Furthermore, T_2_Prep‐IR BOOST led to improved visible vessel length for the RCA, whereas visible LAD vessel length was comparable to that of the dedicated CMRA. In this study, the collection of data with the BOOST and dedicated CMRA sequences always was performed sequentially, without randomizing the order of the two acquisitions. Consequently, irregularities in the respiratory motion pattern of the subjects may have occurred due to the extensive scan time, thus having a detrimental effect on the CMRA data. This might be a confounding factor in the higher level of coronary delineation that was quantified with BOOST.

Furthermore, in our sequence implementation, the iNAV is acquired with high–low profile order, whereas imaging data collection is performed with low–high profile order to minimize the time between the iNAV and imaging to allow for reliable motion estimation. To achieve high SNR required for reliable motion tracking, the iNAV always was acquired with a high flip angle (Fig. 4), which resulted in a high‐flip angle reference scan (T_2_Prep BOOST) to preserve the steady‐state condition. However, the use of high flip‐angle imaging pulses, together with T_2_ preparation in even heartbeats, may impact magnetization recovery and thus diminish the imaging signal in odd heartbeats. Despite this fact, we observed improved CNR_blood‐myo_ when comparing dedicated CMRA and T_2_Prep‐IR BOOST. Further studies are warranted to corroborate these findings and assess the diagnostic accuracy of T_2_Prep‐IR BOOST in comparison to more conventional and dedicated angiography sequences.

Feasibility for thrombus detection with the proposed approach was preliminary demonstrated in an ex vivo pig heart presenting a blood clot. The black‐blood BOOST image showed effective thrombus depiction, with high contrast between the signal from both the myocardium and the cavities.

### Study Limitations

The work presented in this paper has several limitations. First, the motion correction algorithm used in this study approximates the displacement of the heart due to respiration as pure translational motion along the SI and LR direction to facilitate fast inline reconstruction. It is known, however, that the respiratory‐induced displacement of the heart has a much more complex pattern, also involving translations along the anterior–posterior (AP) direction, rotations, nonrigid deformations 38, 39, 40, and hysteresis phenomena 41. Although the approximation of such a motion as pure 2D translation might be a reasonable assumption in most cases—and proved to lead to effective respiratory motion compensation in our healthy volunteer cohort—this could be insufficient in patients, where respiratory motion usually is more complex and irregular 18. In addition, the first acquired iNAV was used as respiratory reference position for translational motion correction; however, this might be suboptimal depending on the respiratory phase during which the scan was started. Previous studies report effective strategies to identify optimized respiratory reference positions 18, 42 and might be beneficial to the proposed framework in future patient studies.

Furthermore, the sequence introduced in this study requires prolonged acquisition times (∼18 minutes) because two high‐resolution bright‐blood 3D datasets are acquired. However, a third black‐blood dataset carrying complementary diagnostic information can be obtained during postprocessing, which may justify the longer scan time. Additionally, the scan time is comparable to a diaphragmatic navigator gated CMRA sequence with the same spatial resolution and volumetric coverage with ∼50% scan efficiency, and is about two times faster than a diaphragmatic navigator gated conventional PSIR acquisition with the same spatial resolution and volumetric coverage with ∼50% scan efficiency. The use of parallel imaging 43, 44, 45 or compressed sensing reconstruction 46 may help further reduce the imaging time to significantly less than 10 minutes.

Feasibility for thrombus detection was demonstrated by imaging an ex vivo pig heart with a ventricular blood clot. In our ex vivo study, the measured T_1_ relaxation time of the myocardium was lower than what it is normally measured in vivo 47; however, effects of formalin on myocardial T_1_ shortening have been reported in previous literature 48, 49. Nevertheless, thrombus T_1_ still was significantly lower than that of myocardium, as reported in previous studies 50, 51, and thus was readily visible on the PSIR images.

### Future Perspectives

The BOOST sequence proposed in this work showed promising results for both bright‐blood and black‐blood coronary artery imaging and now warrants corroborating our preliminary findings in patients with coronary thrombus, complex plaque, or intraplaque hemorrhage. In addition, the suitability of the proposed BOOST framework for coronary plaque characterization in concert with contrast agents needs to be investigated in patients with stable and unstable angina 52. Furthermore, the T_2_Prep‐IR bright‐blood volumes obtained with the proposed BOOST sequence showed improved coronary %VS and CNR between blood and myocardium in comparison to a conventional CMRA acquisition. Therefore, investigation of the diagnostic value of BOOST in patients with known CAD also is warranted to fully assess its robustness and reliability for coronary lumen visualization.

Future technical developments will include the integration of the proposed BOOST framework with acceleration techniques and strategies for nonrigid motion correction to speed up acquisition times and refine the process of motion detection and correction. Motion correction could be implemented in two steps, as proposed in 17, with 1) beat‐to‐beat translational motion correction performed inline in the MR scanner software (∼60 s reconstruction time) and 2) nonrigid motion correction performed inline or offline but requiring a longer reconstruction time (∼1 hour in conventional Central Processing Unit computer).

## CONCLUSION

This study introduces a 3D whole‐heart BOOST sequence for noncontrast enhanced and simultaneous bright‐blood and black‐blood coronary MR imaging for the depiction of coronary lumen and intraluminal thrombosis/intraplaque hemorrhage. The framework includes 2D image‐based navigation to ensure 100% scan efficiency as well as predictable scan time. The proposed approach provided improved coronary lumen visualization when compared to a dedicated CMRA sequence and proved feasibility for thrombus depiction in an ex vivo pig heart. The BOOST framework holds promises for comprehensive assessment of CAD and will be further validated in dedicated clinical studies.

## Supporting information

Additional supporting information may be found in the online version of this article.

**Table S1**. Numerical results from the phantom acquisitions. Data were acquired using a conventional PSIR sequence (22), a modified version of it (23) where a T2Prep‐IR module is applied at even heartbeats, and the proposed BOOST sequence. For both odd and even heartbeats, SNR of blood and CNR between blood and myocardium are reported. For the images obtained after PSIR reconstruction, CNR between blood and myocardium as well as between thrombus and blood are reported.

## References

[1] Libby P . Current concepts of the pathogenesis of the acute coronary syndromes. Circulation 2001;104:365–372. 11457759 10.1161/01.cir.104.3.365

[2] Glagov S , Weisenberg E , Zarins CK , Stankunavicius R , Kolettis GJ . Compensatory enlargement of human atherosclerotic coronary arteries. N Engl J Med 1987;316:1371–1375. 3574413 10.1056/NEJM198705283162204

[3] Noguchi T , Kawasaki T , Tanaka A , Yasuda S , Goto Y , Ishihara M , Nishimura K , Miyamoto Y , Node K , Koga N . High‐intensity signals in coronary plaques on noncontrast T1‐weighted magnetic resonance imaging as a novel determinant of coronary events. J Am Coll Cardiol 2014;63:989–999. 24345595 10.1016/j.jacc.2013.11.034

[4] Kim WY , Danias PG , Stuber M , et al. Coronary magnetic resonance angiography for the detection of coronary stenoses. N Engl J Med 2001;345:1863–1869. 11756576 10.1056/NEJMoa010866

[5] Jansen CH , Perera D , Makowski MR , et al. Detection of intracoronary thrombus by magnetic resonance imaging in patients with acute myocardial infarction. Circulation 2011;124:416–424. 21747055 10.1161/CIRCULATIONAHA.110.965442

[6] Maintz D , Ozgun M , Hoffmeier A , Fischbach R , Kim WY , Stuber M , Manning WJ , Heindel W , Botnar RM . Selective coronary artery plaque visualization and differentiation by contrast‐enhanced inversion prepared MRI. Eur Heart J 2006;27:1732–1736. 16787955 10.1093/eurheartj/ehl102

[7] Kawasaki T , Koga S , Koga N , et al. Characterization of hyperintense plaque with noncontrast T(1)‐weighted cardiac magnetic resonance coronary plaque imaging: comparison with multislice computed tomography and intravascular ultrasound. JACC Cardiovasc Imaging 2009;2:720–728. 19520342 10.1016/j.jcmg.2009.01.016

[8] Ehman RL , Felmlee JP . Adaptive technique for high‐definition MR imaging of moving structures. Radiology 1989;173:255–263. 2781017 10.1148/radiology.173.1.2781017

[9] Stehning C , Bornert P , Nehrke K , Eggers H , Stuber M . Free‐breathing whole‐heart coronary MRA with 3D radial SSFP and self‐navigated image reconstruction. Magn Reson Med 2005;54:476–480. 16032682 10.1002/mrm.20557

[10] Lai P , Bi X , Jerecic R , Li D . A respiratory self‐gating technique with 3D‐translation compensation for free‐breathing whole‐heart coronary MRA. Magn Reson Med 2009;62:731–738. 19526514 10.1002/mrm.22058 PMC4426993

[11] Piccini D , Littmann A , Nielles‐Vallespin S , Zenge MO . Respiratory self‐navigation for whole‐heart bright‐blood coronary MRI: methods for robust isolation and automatic segmentation of the blood pool. Magn Reson Med 2012;68:571–579. 22213169 10.1002/mrm.23247

[12] Henningsson M , Koken P , Stehning C , Razavi R , Prieto C , Botnar RM . Whole‐heart coronary MR angiography with 2D self‐navigated image reconstruction. Magn Reson Med 2012;67:437–445. 21656563 10.1002/mrm.23027

[13] Pang J , Bhat H , Sharif B , Fan Z , Thomson LE , LaBounty T , Friedman JD , Min J , Berman DS , Li D . Whole‐heart coronary MRA with 100% respiratory gating efficiency: self‐navigated three‐dimensional retrospective image‐based motion correction (TRIM). Magn Reson Med 2014;71:67–74. 23401157 10.1002/mrm.24628 PMC3655135

[14] Ingle RR , Wu HH , Addy NO , Cheng JY , Yang PC , Hu BS , Nishimura DG . Nonrigid autofocus motion correction for coronary MR angiography with a 3D cones trajectory. Magn Reson Med 2014;72:347–361. 24006292 10.1002/mrm.24924 PMC3942375

[15] Prieto C , Doneva M , Usman M , Henningsson M , Greil G , Schaeffter T , Botnar RM . Highly efficient respiratory motion compensated free‐breathing coronary MRA using golden‐step Cartesian acquisition. J Magn Reson Imaging 2015;41:738–746. 24573992 10.1002/jmri.24602

[16] Aitken AP , Henningsson M , Botnar RM , Schaeffter T , Prieto C . 100% efficient three‐dimensional coronary MR angiography with two‐dimensional beat‐to‐beat translational and bin‐to‐bin affine motion correction. Magn Reson Med 2015;74:756–764. 25236813 10.1002/mrm.25460

[17] Cruz G , Atkinson D , Henningsson M , Botnar RM , Prieto C . Highly efficient nonrigid motion‐corrected 3D whole‐heart coronary vessel wall imaging. Magn Reson Med 2017;77:1894–1908. 27221073 10.1002/mrm.26274 PMC5412916

[18] Ginami G , Bonanno G , Schwitter J , Stuber M , Piccini D . An iterative approach to respiratory self‐navigated whole‐heart coronary MRA significantly improves image quality in a preliminary patient study. Magn Reson Med 2016;75:1594–1604. 25960337 10.1002/mrm.25761

[19] Luo J , Addy NO , Ingle RR , Baron CA , Cheng JY , Hu BS , Nishimura DG . Nonrigid motion correction with 3D image‐based navigators for coronary MR angiography. Magn Reson Med 2017;77:1884–1893. 27174673 10.1002/mrm.26273 PMC5107365

[20] Matsumoto K , Ehara S , Hasegawa T , Sakaguchi M , Otsuka K , Yoshikawa J , Shimada K . Localization of coronary high‐intensity signals on T1‐weighted MR imaging: relation to plaque morphology and clinical severity of angina pectoris. JACC Cardiovasc Imaging 2015;8:1143–1152. 26363839 10.1016/j.jcmg.2015.06.013

[21] Xie Y , Kim YJ , Pang J , et al. Coronary atherosclerosis T1‐weighed characterization with integrated anatomical reference: comparison with high‐risk plaque features detected by invasive coronary imaging. JACC Cardiovasc Imaging 2017;10:637–648. 27743950 10.1016/j.jcmg.2016.06.014 PMC7388693

[22] Kellman P , Arai AE , McVeigh ER , Aletras AH . Phase‐sensitive inversion recovery for detecting myocardial infarction using gadolinium‐delayed hyperenhancement. Magn Reson Med 2002;47:372–383. 11810682 10.1002/mrm.10051 PMC2041905

[23] Xie J , Bi X , Fan Z , Bhat H , Shah S , Zuehlsdorff S , Li D . 3D flow‐independent peripheral vessel wall imaging using T(2)‐prepared phase‐sensitive inversion‐recovery steady‐state free precession. J Magn Reson Imaging 2010;32:399–408. 20677269 10.1002/jmri.22272 PMC2915467

[24] Atlas SW , Grossman RI , Hackney DB , Goldberg HI , Bilaniuk LT , Zimmerman RA . STIR MR imaging of the orbit. AJR Am J Roentgenol 1988;151:1025–1030. 3263000 10.2214/ajr.151.5.1025

[25] Haase A , Frahm J , Hanicke W , Matthaei D . 1H NMR chemical shift selective (CHESS) imaging. Phys Med Biol 1985;30:341–344. 4001160 10.1088/0031-9155/30/4/008

[26] Sussman MS , Wright GA . Factors affecting the correlation coefficient template matching algorithm with application to real‐time 2‐D coronary artery MR imaging. IEEE Trans Med Imaging 2003;22:206–216. 12715997 10.1109/TMI.2002.808363

[27] Weigel M . Extended phase graphs: dephasing, RF pulses, and echoes—pure and simple. J Magn Reson Imaging 2015;41:266–295. 24737382 10.1002/jmri.24619

[28] Captur G , Gatehouse P , Keenan KE , et al. A medical device‐grade T1 and ECV phantom for global T1 mapping quality assurance‐the T1 mapping and ECV standardization in cardiovascular magnetic resonance (T1MES) program. J Cardiovasc Magn Reson 2016;18:58. 27660042 10.1186/s12968-016-0280-z PMC5034411

[29] De Wilde JP , Lunt JA , Straughan K . Information in magnetic resonance images: evaluation of signal, noise and contrast. Med Biol Eng Comput 1997;35:259–265. 9246861 10.1007/BF02530047

[30] Etienne A , Botnar RM , Van Muiswinkel AM , Boesiger P , Manning WJ , Stuber M . “Soap‐Bubble” visualization and quantitative analysis of 3D coronary magnetic resonance angiograms. Magn Reson Med 2002;48:658–666. 12353283 10.1002/mrm.10253

[31] Messroghli DR , Radjenovic A , Kozerke S , Higgins DM , Sivananthan MU , Ridgway JP . Modified look‐locker inversion recovery (MOLLI) for high‐resolution T1 mapping of the heart. Magn Reson Med 2004;52:141–146. 15236377 10.1002/mrm.20110

[32] Giri S , Chung YC , Merchant A , Mihai G , Rajagopalan S , Raman SV , Simonetti OP . T2 quantification for improved detection of myocardial edema. J Cardiovasc Magn Reson 2009;11:56. 20042111 10.1186/1532-429X-11-56 PMC2809052

[33] Kino A , Zuehlsdorff S , Sheehan JJ , Weale PJ , Carroll TJ , Jerecic R , Carr JC . Three‐dimensional phase‐sensitive inversion‐recovery turbo FLASH sequence for the evaluation of left ventricular myocardial scar. AJR Am J Roentgenol 2009;193:W381–W388. 19843715 10.2214/AJR.08.1952

[34] Kino A , Keeling AN , Farrelly CT , Sheehan JJ , Davarpanah AH , Weele PJ , Zuehldorff S , Carr JC . Assessment of left ventricular myocardial scar in infiltrative and non‐ischemic cardiac diseases by free breathing three‐dimensional phase sensitive inversion recovery (PSIR) TurboFLASH. Int J Cardiovasc Imaging 2011;27:527–537. 20499279 10.1007/s10554-010-9640-1

[35] Kido T , Kido T , Nakamura M , Kawaguchi N , Nishiyama Y , Ogimoto A , Miyagawa M , Mochizuki T . Three‐dimensional phase‐sensitive inversion recovery sequencing in the evaluation of left ventricular myocardial scars in ischemic and non‐ischemic cardiomyopathy: comparison to three‐dimensional inversion recovery sequencing. Eur J Radiol 2014;83:2159–2166. 25311877 10.1016/j.ejrad.2014.09.014

[36] Abd‐Elmoniem KZ , Weiss RG , Stuber M . Phase‐sensitive black‐blood coronary vessel wall imaging. Magn Reson Med 2010;63:1021–1030. 20373403 10.1002/mrm.22286

[37] Ginami G , Yerly J , Masci PG , Stuber M . Golden angle dual‐inversion recovery acquisition coupled with a flexible time‐resolved sparse reconstruction facilitates sequence timing in high‐resolution coronary vessel wall MRI at 3 T. Magn Reson Med 2017;77:961–969. 26900941 10.1002/mrm.26171

[38] Manke D , Nehrke K , Bornert P , Rosch P , Dossel O . Respiratory motion in coronary magnetic resonance angiography: a comparison of different motion models. J Magn Reson Imaging 2002;15:661–671. 12112516 10.1002/jmri.10112

[39] Manke D , Nehrke K , Bornert P . Novel prospective respiratory motion correction approach for free‐breathing coronary MR angiography using a patient‐adapted affine motion model. Magn Reson Med 2003;50:122–131. 12815687 10.1002/mrm.10483

[40] Shechter G , Ozturk C , Resar JR , McVeigh ER . Respiratory motion of the heart from free breathing coronary angiograms. IEEE Trans Med Imaging 2004;23:1046–1056. 15338737 10.1109/TMI.2004.828676 PMC2494710

[41] Nehrke K , Bornert P , Manke D , Bock JC . Free‐breathing cardiac MR imaging: study of implications of respiratory motion—initial results. Radiology 2001;220:810–815. 11526286 10.1148/radiol.2203010132

[42] Piccini D , Bonanno G , Ginami G , Littmann A , Zenge MO , Stuber M . Is there an optimal respiratory reference position for self‐navigated whole‐heart coronary MR angiography? J Magn Reson Imaging 2016;43:426–433. 26174582 10.1002/jmri.24992

[43] Sodickson DK , Manning WJ . Simultaneous acquisition of spatial harmonics (SMASH): fast imaging with radiofrequency coil arrays. Magn Reson Med 1997;38:591–603. 9324327 10.1002/mrm.1910380414

[44] Pruessmann KP , Weiger M , Scheidegger MB , Boesiger P . SENSE: sensitivity encoding for fast MRI. Magn Reson Med 1999;42:952–962. 10542355

[45] Griswold MA , Jakob PM , Heidemann RM , Nittka M , Jellus V , Wang J , Kiefer B , Haase A . Generalized autocalibrating partially parallel acquisitions (GRAPPA). Magn Reson Med 2002;47:1202–1210. 12111967 10.1002/mrm.10171

[46] Lustig M , Donoho D , Pauly JM . Sparse MRI: the application of compressed sensing for rapid MR imaging. Magn Reson Med 2007;58:1182–1195. 17969013 10.1002/mrm.21391

[47] Brittain JH , Hu BS , Wright GA , Meyer CH , Macovski A , Nishimura DG . Coronary angiography with magnetization‐prepared T2 contrast. Magn Reson Med 1995;33:689–696. 7596274 10.1002/mrm.1910330515

[48] Carpenter JP , He T , Kirk P , et al. Calibration of myocardial T2 and T1 against iron concentration. J Cardiovasc Magn Reson 2014;16:62. 25158620 10.1186/s12968-014-0062-4 PMC4145261

[49] Fishbein KW , Gluzband YA , Kaku M , Ambia‐Sobhan H , Shapses SA , Yamauchi M , Spencer RG . Effects of formalin fixation and collagen cross‐linking on T2 and magnetization transfer in bovine nasal cartilage. Magn Reson Med 2007;57:1000–1011. 17534923 10.1002/mrm.21216

[50] Saha P , Andia ME , Modarai B , et al. Magnetic resonance T1 relaxation time of venous thrombus is determined by iron processing and predicts susceptibility to lysis. Circulation 2013;128:729–736. 23820077 10.1161/CIRCULATIONAHA.113.001371 PMC3983557

[51] Andia ME , Saha P , Jenkins J , Modarai B , Wiethoff AJ , Phinikaridou A , Grover SP , Patel AS , Schaeffter T , Smith A , Botnar RM . Fibrin‐targeted magnetic resonance imaging allows in vivo quantification of thrombus fibrin content and identifies thrombi amenable for thrombolysis. Arterioscler Thromb Vasc Biol 2014;34:1193–1198. 24723557 10.1161/ATVBAHA.113.302931 PMC4195984

[52] Botnar RM , Ebersberger H , Noerenberg D , et al. Molecular imaging in cardiovascular diseases. Rofo 2015;36:92–101. 25912326 10.1055/s-0034-1385451

