# Supplementary material for: Simultaneous bright‐ and black‐blood whole‐heart MRI for noncontrast enhanced coronary lumen and thrombus visualization
Source: Magn Reson Med. 2017 Jul 19;79(3):1460–72. doi: 10.1002/mrm.26815 (PMC5811778; doi:10.1002/mrm.26815)
Supplement: Supplementary file 1 — Table S1. Numerical results from the phantom acquisitions. Data were acquired using a conventional PSIR sequence (22), a modified version of it (23) where a T2Prep‐IR module is applied at even heartbeats, and the proposed BOOST sequence. For both odd and even heartbeats, SNR of blood and CNR between blood and myocardium are reported. For the images obtained after PSIR reconstruction, CNR between blood and myocardium as well as between thrombus and blood are reported. [file MRM-79-1460-s001.pdf]

SUPPORTING INFORMATION

	Odd heartbeats		Even heartbeats		PSIR	
	1	2	1	2	2	3
**Conventional PSIR**	81.88	28.44	5.6	10.02	~0	~0
**T2 Prep – IR PSIR**	61.25	27.42	7.46	10.7	27.9	52.6
**BOOST**	61.6	31.06	33.18	8.28	46.46	58.04

1: SNR blood.
2: CNR blood-myocardium.
3: CNR thrombus- blood.

Table S1: Numerical results from the phantom acquisitions. Data were acquired using a conventional PSIR sequence (22), a modified version of it (23) where a T2Prep-IR module is applied at even heartbeats, and the proposed BOOST sequence. For both odd and even heartbeats, SNR of blood and CNR between blood and myocardium are reported. For the images obtained after PSIR reconstruction, CNR between blood and myocardium as well as between thrombus and blood are reported.
